# Differential Rates of Early Gastric Cancer in the Urban and Rural Medical Centers of Hangzhou, China

**DOI:** 10.14309/ctg.0000000000000851

**Published:** 2025-05-06

**Authors:** Lu Sun, Qingfeng Yang, Bin Lyu, Yanjie Shen, Yeqi He, Yi Zhang, Liang Han

**Affiliations:** 1 Department of Gastroenterology, Hangzhou Ninth People's Hospital (Zhejiang Hospital of Integrated Traditional Chinese and Western Medicine Qiantang Campus), Hangzhou, China;; 2 Department of Gastroenterology, First Affiliated Hospital of Zhejiang Chinese Medical University, Hangzhou, China;; 3 Key Laboratory of Digestive Pathophysiology of Zhejiang Province, First Affiliated Hospital of Zhejiang Chinese Medical University, Hangzhou, China;; 4 Department of Pathology, Hangzhou Ninth People's Hospital (Zhejiang Hospital of Integrated Traditional Chinese and Western Medicine Qiantang Campus), Hangzhou, China.

**Keywords:** gastric cancer, endoscopy, early cancer detection, rural health, atrophic gastritis

## Abstract

**INTRODUCTION:**

The aim of this study was to compare gastric cancer (GC) and early GC (EGC) diagnosis rates between urban and rural and to investigate potential reasons for the increased GC morbidity in rural areas.

**METHODS:**

Patients who underwent endoscopy at rural and urban medical centers from 2019 to 2024 were included. We analyzed differences in patients' pre-endoscopic chief complaints and endoscopic diagnoses across the 2 areas.

**RESULTS:**

Thirty-two thousand six hundred thirteen patients from rural medical centers and 70,195 patients from urban centers were included. Significant differences in endoscopic diagnoses were found between the groups, with the EGC diagnosis rate being significantly lower in rural areas than in urban (10.19% vs. 27.19%). Rural patients were more likely to undergo endoscopy for abdominal pain, reflux, abdominal fullness, and melena (relative risk [RR] = 1.340, 1.431, 1.106, and 1.231, respectively). Fewer rural patients underwent endoscopy because of laboratory abnormality, including *Helicobacter pylori* infection, elevated tumor markers, positive fecal occult blood tests, and anemia (RR = 0.591, 0.295, 0.251, and 0.400, respectively). In addition, rural patients were significantly less likely to undergo endoscopy owing to health screening or surveillance for chronic atrophic gastritis (RR = 0.362 and 0.527, respectively).

**DISCUSSION:**

The diagnosis rate of EGC is significantly lower in rural than in urban. Rural patients are more likely to seek endoscopy because they are symptomatic and are less likely to undergo endoscopy for health screening, surveillance for chronic atrophic gastritis, or laboratory abnormality. Enhanced health education and awareness programs in rural areas are needed to encourage proactive endoscopic screening and surveillance.

## Graphical abstract

**Figure:**
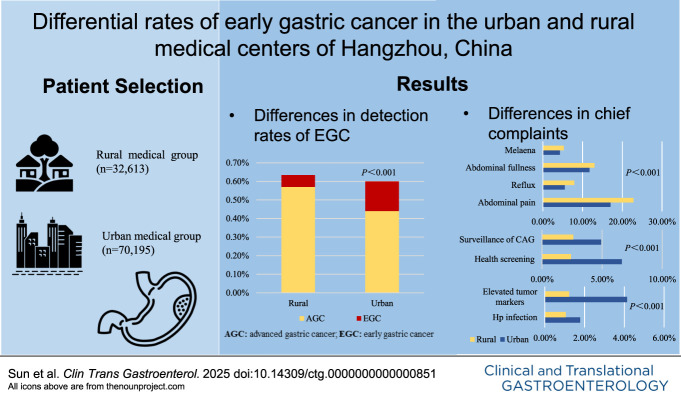

## INTRODUCTION

The upper gastrointestinal tract is affected by numerous common digestive diseases, including gastric precancerous conditions and gastric cancer (GC). Endoscopy is a crucial diagnostic tool for upper gastrointestinal diseases and is widely used for the diagnosis and management of precancerous gastric conditions and GC.

Among all cancers, GC, the fifth most common cancer globally, ranks fourth in mortality (1). The prognosis for GC is closely linked to its stage; the 5-year survival rate for advanced gastric cancer (AGC) is only 10%–30% (2), whereas for early gastric cancer (EGC), it exceeds 90% (3). Thus, early diagnosis is critical for GC prevention and treatment. Chronic atrophic gastritis (CAG) is a precancerous condition of the stomach (4), and endoscopic surveillance of CAG can facilitate the early diagnosis of GC, significantly improving patient outcomes (5).

In recent years, with the rapid development of endoscopic technology and the widespread popularization of patients' disease and health awareness, the rate of early diagnosis and treatment of GC in some developed areas, such as Japan and South Korea, can reach 50%–60%. However, in China, the early diagnosis rate remains below 20% (6), particularly in rural areas, where GC incidence and mortality are higher than those in urban areas (7), and early diagnosis rates are even lower. Improving the early diagnosis rate of GC in rural areas is a critical issue that needs to be solved. Owing to the frequently asymptomatic nature of EGC, many patients miss the opportunity for early treatment, seeking medical attention only when AGC symptoms develop (8). In previous studies on the early diagnosis of GC, more attention has been devoted to physician practices and endoscopy equipment, but less to the patients themselves. Patients’ chief complaints lead them to undergo endoscopy, which reflects their awareness of early disease diagnosis and treatment and also suggests the disease status accordingly. Studying the differences in pre-endoscopy complaints and endoscopic diagnosis between urban and rural patients is of great clinical and public health value. Identifying gaps in rural patients' awareness of early diagnosis and treatment can help inform targeted medical interventions and health education efforts, ultimately increasing EGC diagnosis rates, reducing healthcare costs, and improving patient outcomes.

The aim of this study was to explore the differences in the early diagnosis rates of GC and chief complaints between urban and rural patients in Hangzhou, China. By examining these differences, we seek to guide the development of health education programs and promote endoscopy in rural areas.

## MATERIALS AND METHODS

### Patient selection

This study included patients who underwent upper gastrointestinal endoscopy at a rural medical center (Hangzhou Ninth People's Hospital) and an urban medical center (First Affiliated Hospital of Zhejiang Chinese Medical University) in Hangzhou, China, from January 1, 2019, to August 31, 2024. Rural means locating outside cities and towns. Urban means belonging to, or relating to, a city or town. Both centers are upper-level hospitals, and both are medical centers in their locations. Both centers used Olympus (GIF-260 and GIF-290) endoscopic equipment.

The exclusion criteria were as follows: (i) incomplete medical history data or missing chief complaint data, (ii) missing endoscopic or pathological data, and (iii) a prior confirmed diagnosis of GC from a lower-level hospital with a referral to the medical center.

### Data collection

Demographic information: Demographic data, including sex, age, and examination date, were collected for all included patients.

Medical history: Patients' chief complaints before endoscopy were recorded and categorized as follows:

1. Symptom category: Symptoms associated with GC (8), including abdominal pain, reflux, nausea and vomiting, abdominal fullness, melena, early satiety, and poor appetite.
2. Laboratory abnormality: Abnormalities detected before endoscopy, including positive *Helicobacter pylori* infection, elevated tumor markers, positive fecal occult blood, and anemia. The above laboratory tests are widely available in both rural and urban areas of Hangzhou.
3. Physical examination category: Routine health checkups.
4. Disease monitoring category: Surveillance for precancerous gastric conditions, specifically CAG.

Multiple chief complaints presented in a patient were all documented:

#### Endoscopic data.

1. Endoscopic diagnoses: The diagnoses were confirmed through pathology.
2. For EGC cases, Paris classification staging (0-I, 0-IIa, 0-IIb, 0-IIc, 0-III) was recorded, with mixed staging recorded with the dominant lesion pattern.
3. For AGC cases, the Borrmann classification (I-IV) was documented.
4. Documentation of other disease diagnoses: Multiple diagnoses for the same patient were all recorded.

#### Pathological data.

1. Histopathologic diagnoses of mucosal biopsies, including atrophy, intestinal metaplasia, active inflammation, chronic inflammation, and *H. pylori* infection, using the new Sydney system.
2. Pathological reports of biopsies of the mucosal lesion and resection of the lesion were all recorded.
3. EGC resected by endoscopic submucosal dissection (ESD), with documentation of gastric carcinoma size, location, morphology, differentiation, depth of infiltration, and lateral and deep margin status, and with recorded R0 resection.

GC diagnoses at both centers were based on Japanese criteria (9), defining EGC as GC limited to the mucosa or submucosa, with or without lymph node involvement. Mucosal biopsy pathology was performed routinely during endoscopy, with additional biopsies for suspicious lesions. All resected lesions were subjected to pathological analysis. R0 resection was defined as the absence of tumor cells at both the lateral and deep resection margins. The endoscopists at both centers are professionally trained and highly experienced in diagnostic endoscopy.

### Research methods

Patients were selected based on inclusion and exclusion criteria and categorized into 2 cohorts according to their medical center.

1. Rural medical group: Patients who attended a rural medical center and underwent endoscopy.
2. Urban medical group: Patients who were admitted to an urban medical center and underwent endoscopy.

The study focused on analyzing the following data:

1. Differences in endoscopic diagnoses between the 2 groups.
2. Differences in chief complaints before endoscopy.
3. Differences in endoscopic biopsy pathology, resected lesions, and GC lesions between the 2 groups.

**Figure 1.**
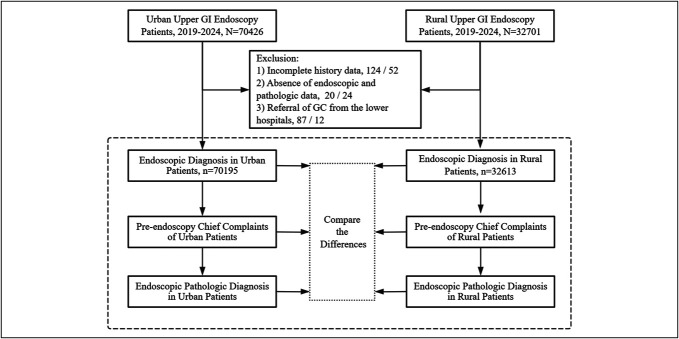
Flowchart of the study. GC, gastric cancer; GI, gastrointestinal.

The study flowchart is illustrated in Figure 1.

### Statistical analysis

The data were analyzed using SPSS 25.0. For normally distributed variables, data are expressed as the mean ± SD; for skewed data, data are expressed as the median with interquartile range. Rates and ratios are expressed as N (%). The χ^2^ test and/or the Fisher exact test were used to compare differences in GC detection, including chief complaints, endoscopic diagnoses, GC diagnosis rate, GC stage, resection status, pathological diagnosis, and *H. pylori* positivity rate, between urban and rural patients. A 2-sided *P* value of < 0.05 was considered statistically significant. The relative risk (RR) was calculated to assess the risk or protection.

This retrospective cohort study was conducted according to the Declaration of Helsinki, and all data were anonymized for research purposes only. This study was approved by the Clinical Ethics Committee of the institution (Approval number: 2024-078).

## RESULTS

A total of 102,808 patients with complete medical histories and endoscopic and pathological data were included, with 32,613 patients in the rural group (mean age 49.21 ± 15.00 years, 53.53% male) and 70,195 in the urban group (mean age 50.45 ± 15.12 years, 52.74% male). In urban medical centers, senior physicians performed 65.80% (46,187/70,195) of endoscopic procedures, whereas in rural medical centers, senior physicians conducted 66.64% (21,738/32,613) of procedures.

**Figure 2.**
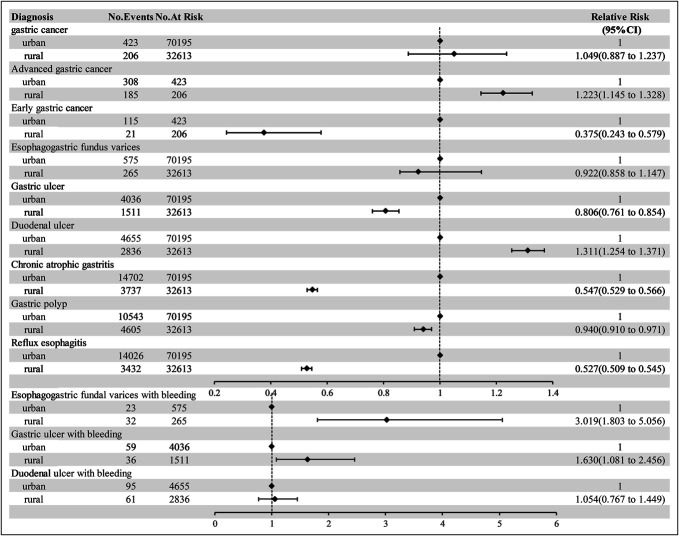
Endoscopic diagnosis and relative risk analysis between the 2 groups.

Endoscopic reports identified 629 GC cases across both centers: 206 in rural centers and 423 in urban centers, with no statistically significant difference in GC detection rates. However, advanced gastric cancer was significantly more prevalent among rural patients than among urban patients (89.81% vs. 72.81%, *P* < 0.001, RR = 1.223). The EGC diagnosis rate in rural areas was markedly low (10.19%), with 21 cases comprising 22 lesions (20 single lesions and 1 case with 2 lesions). By contrast, urban areas had an EGC diagnosis rate of 27.19%, with 115 cases comprising 121 lesions (109 single lesions and 6 cases with 2 lesions). Rural patients also had a significantly greater prevalence of duodenal ulcers, bleeding esophagogastric varices, and bleeding gastric ulcers, whereas urban patients had higher rates of reflux esophagitis and CAG. Details are provided in Table 1 and Figure 2.

**Table 1.** Demographic characteristics and endoscopic diagnoses of the included patients

Characteristics and diagnose	Rural medical group = 32,613	Urban medical group = 70,195	*P*
Sex			0.018
Male	17,459 (53.53%)	37,024 (52.74%)	
Female	15,154 (46.47%)	33,171 (47.26%)	
Age (Mean ± SD)	49.21 ± 15.00	50.45 ± 15.12	<0.001
Endoscopic diagnoses			
GC	206 (0.63%)	423 (0.60%)	0.578
EGC	20 + 1 × 2 (10.19%)	109 + 6 × 2 (27.19%)	<0.001
IIa	11	59	
IIb	2	13	
IIc	9	49	
AGC	185 (89.81%)	308 (72.81%)	<0.001
Borrmann I	30	71	
Borrmann II	61	66	
Borrmann III	84	143	
Borrmann IV	10	28	
Esophagogastric varices	265 (0.81%)	575 (0.82%)	0.913
With bleeding	32 (12.08%)	23 (4.00%)	<0.001
Without bleeding	233 (87.92%)	552 (96.00%)	
Gastric ulcers	1,511 (4.63%)	4,036 (5.75%)	<0.001
With bleeding	36 (2.38%)	59 (1.46%)	0.019
Without bleeding	1,475 (97.62%)	3,977 (98.54%)	
Duodenal ulcers	2,836 (8.70%)	4,655 (6.63%)	<0.001
With bleeding	61 (2.15%)	95 (2.04%)	0.746
Without bleeding	2,775 (97.85%)	4,560 (97.96%)	
CAG	3,737 (11.46%)	14,702 (20.94%)	<0.001
Gastric polyp	4,605 (14.12%)	10,543 (15.02%)	<0.001
Reflux esophagitis	3,432 (10.52%)	14,026 (19.98%)	<0.001

AGC, advanced gastric cancer; CAG, chronic atrophic gastritis; EGC, early gastric cancer; GC, gastric cancer.

**Figure 3.**
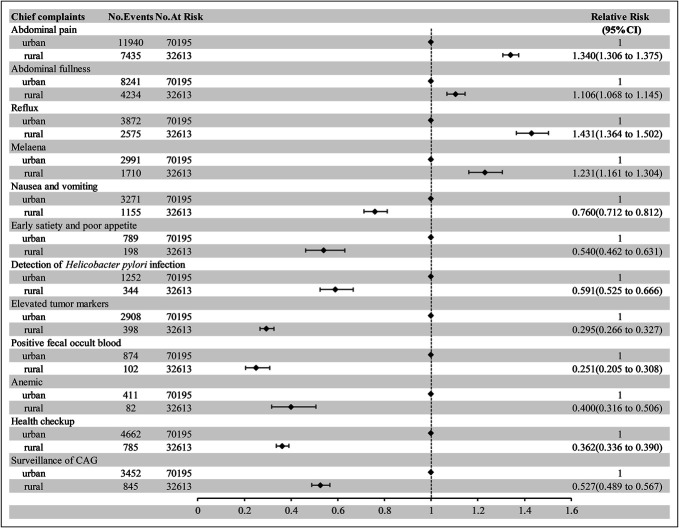
Pre-endoscopic chief complaint rate and relative risk analysis between the 2 groups. CAG, chronic atrophic gastritis.

Further analysis of pre-endoscopy chief complaints revealed that rural patients were more likely to undergo endoscopy with symptoms such as abdominal pain, reflux, abdominal fullness, and melena than urban patients were (all *P* < 0.001, RR = 1.340, 1.431, 1.106, and 1.231, respectively). Conversely, rural patients had significantly lower rates of endoscopy for laboratory abnormality, such as *H. pylori* infection, elevated tumor markers, positive fecal occult blood, and anemia, than did urban patients (all *P* < 0.001, RR = 0.591, 0.295, 0.251, 0.400, respectively). Fewer rural patients than urban patients underwent gastroscopy for routine health checkups or CAG surveillance (all *P* < 0.001, RR = 0.362, 0.527, respectively). Details are presented in Table 2 and Figure 3.

**Table 2.** Pre-endoscopy chief complaints of the included patients

Chief complaints	Rural medical group = 32,613	Urban medical group = 70,195	*P*
Symptom category			
Abdominal pain	7,435 (22.80%)	11,940 (17.01%)	<0.001
Reflux	2,575 (7.90%)	3,872 (5.52%)	<0.001
Nausea and vomiting	1,155 (3.54%)	3,271 (4.66%)	<0.001
Abdominal fullness	4,234 (12.98%)	8,241 (11.74%)	<0.001
Melena	1710 (5.24%)	2,991 (4.26%)	<0.001
Early satiety and poor appetite	198 (0.61%)	789 (1.12%)	<0.001
Laboratory abnormality			
*Helicobacter pylori* infection	344 (1.05%)	1,252 (1.78%)	<0.001
Elevated tumor markers	398 (1.22%)	2,908 (4.14%)	<0.001
Positive fecal occult blood	102 (0.31%)	874 (1.25%)	<0.001
Anemic	82 (0.25%)	441 (0.63%)	<0.001
Physical examination			
Health checkup	785 (2.41%)	4,662 (6.64%)	<0.001
Disease monitoring			
Surveillance of CAG	845 (2.60%)	3,452 (4.92%)	<0.001

CAG, chronic atrophic gastritis.

Routine mucosal biopsies and targeted biopsies of suspicious lesions were conducted at both centers. Pathology revealed higher rates of atrophy, intestinal metaplasia, active inflammation, and *H. pylori* positivity in urban patients (all *P* < 0.001), whereas rural patients had a higher rate of chronic inflammation. Among the 136 EGC patients, 14 rural patients (one with 2 lesions, totaling 15 lesions) underwent ESD resection, achieving R0 resection for all lesions. In the urban group, 108 patients (6 with 2 lesions, totaling 114 lesions) underwent ESD resection, with R0 resection achieved for 112 lesions, although 2 patients had positive deep margins. The detailed data are presented in Tables 3 and 4.

**Table 3.** Mucosal biopsy diagnoses of the included patients

New sydney system	Rural medical group	Urban medical group	*P*	RR (95% CI)
Atrophy	2,375 (7.28%)	6,986 (9.95%)	<0.001	0.732 (0.700–0.765)
Intestinal metaplasia	5,967 (18.30%)	19,814 (28.23%)	<0.001	0.648 (0.632–0.665)
Chronic inflammation	30,674 (94.05%)	59,421 (84.65%)	<0.001	1.111 (1.106–1.116)
Active inflammation	4,547 (13.94%)	15,898 (22.65%)	<0.001	0.616 (0.597–0.634)
*Helicobacter pylori*	4,185 (12.83%)	12,628 (17.99%)	<0.001	0.713 (0.691–0.737)

Pathological biopsies were taken from one or more blocks in clinical practice, depending on the patient's endoscopic condition and lesion presentation, and all pathological specimens with the new Sydney system of gastritis were counted here as a percentage of the base number.

CI, confidence interval; RR, relative risk.

**Table 4.** Pathological results of ESD resection for early gastric cancer in the 2 groups

Early gastric cancer	Rural medical group = 13 + 1 × 2	Urban medical group = 102 + 6 × 2
Place of gastric		
Upper 1/3	10	81
Middle 1/3	3	12
Lower 1/3	2	21
Diameter size		
<2 cm	9	93
2–3 cm	4	15
>3 cm	2	6
Paris classification		
Ⅱa	8	56
Ⅱb	2	12
Ⅱc	5	46
Type of differentiation		
Differentiation	15	108
Undifferentiation	0	5
Depth of invasion		
M	14	103
SM1	1	9
SM2	0	2
Lateral margin		
Negative	15	114
Positive	0	0
Deep margin		
Negative	15	112
Positive	0	2
R0 resection		
Yes	15	112
No	0	2

ESD, endoscopic submucosal dissection; M, mucosal layer; SM1, submucosal invasion < 500 μm; SM2, submucosal invasion ≥ 500 μm.

## DISCUSSION

China has one of the highest GC burdens globally, accounting for nearly half of all cases each year (10), posing a substantial public health challenge. Although the overall incidence of GC has been decreasing (11), the incidence and mortality rates remain elevated in rural areas, where early diagnosis rates are particularly low (12). Similarly, rural areas globally face high GC mortality rates and low early detection rates (13–15), highlighting a critical gap in prevention and treatment efforts for rural populations. Enhancing early diagnosis and treatment in rural settings to reduce GC mortality is an urgent issue requiring collaboration between healthcare providers and patients.

With advances in medical technology and the widespread adoption of endoscopic techniques, many primary healthcare facilities in China can now perform endoscopic diagnoses. The average wait time for upper gastrointestinal endoscopy is only 1.36 weeks (16). However, most patients with GC are still diagnosed at an advanced stage, likely due to delays in seeking medical care. GC often presents with minimal or no symptoms in its early stages, complicating early recognition. Compared with urban patients, rural patients are especially prone to delayed diagnoses, with studies indicating that rural populations, particularly elderly individuals, may have limited social resources, lower health literacy, and reduced access to medical screenings (17,18). This often results in delayed consultations, missed opportunities for early diagnosis, and subsequently poorer outcomes. Our findings, which are consistent with those of previous multicenter studies (19), revealed a lower rate of EGC diagnoses in rural patients than in urban patients. This study further investigates the differences in endoscopy-related chief complaints between rural and urban patients from the patients' perspective. Understanding these differences can help primary care physicians better assess the current state of rural health care and improve medical services in these areas. To our knowledge, this is the first study to analyze variations in the purpose of endoscopy between rural and urban patients. Our findings reveal significant deficiencies in endoscopic surveillance and early screening among rural patients.

When the chief complaints before endoscopy were compared, rural patients were more likely than urban patients to report symptomatic concerns such as abdominal pain, reflux, abdominal fullness, and melena. However, rural patients were less likely to pursue endoscopy for reasons such as laboratory abnormality, routine health checkups, or surveillance of CAG. This indicates a gap in asymptomatic screening participation among rural patients, which is a critical barrier in GC prevention and control. Research has shown that the most common reason patients decline endoscopic screening is “no symptoms” (63.0%), followed by “fear of endoscopy” (38.1%), with lower screening acceptance observed in rural populations (20). This suggests that rural patients may be more likely to assume that asymptomatic individuals do not require endoscopic screening. Addressing this misconception will require both robust health education by healthcare providers and enhanced health insurance support from the government to reduce the psychological and financial burden of screening, especially for rural patients whose understanding and financial means are limited. Our study also revealed a greater incidence of peptic ulcers and active bleeding from esophagogastric fundal varices in rural patients, suggesting delayed access to medical care.

Early detection is key to improving GC prognosis, and endoscopy is a crucial method for detecting EGC. Many years of screening in countries such as Japan and South Korea have shown that endoscopic screening significantly increases the EGC diagnosis rate and reduces GC mortality. The risk of death decreases with repeated screenings (21). CAG is a precancerous condition that carries a risk of progression to GC, and recent multinational guidelines recommend regular endoscopic surveillance for individuals with CAG (22,23). Our previous follow-up study of 929 patients with CAG who underwent regular endoscopic surveillance for 36–129 months revealed that 92.9% (24) were diagnosed with EGC and underwent curative endoscopic resection. In addition, our analysis of 1,025 GC cases revealed that lesions detected through endoscopic surveillance were typically smaller, less invasive, and had higher curative resection rates (25). Unfortunately, China lacks a universal endoscopic screening program such as those in Japan and South Korea. In our study, few patients underwent endoscopic screening for routine health checks or surveillance for CAG, with participation rates even lower among rural patients. This may contribute to the lower early diagnosis rates of GC in rural areas. Rural healthcare providers should educate patients about the benefits of endoscopy screening, especially for high-risk groups, and promote long-term surveillance for patients with CAG.

Timely endoscopic screening is essential for patients with laboratory abnormalities, even in the absence of symptoms. Tumor markers, such as carcinoembryonic antigen and carbohydrate antigen 19-9, can aid in the identification and monitoring of gastric adenocarcinoma (26–28), although these markers lack sensitivity and specificity in screening for GC and are not the most effective tools for early detection (29). Nevertheless, abnormalities in these markers may prompt patients to undergo opportunistic endoscopic screening. *H. pylori* is a class I carcinogen and a significant risk factor of GC and precancerous gastric conditions (30). Eradication of *H. pylori* effectively reduces GC risk. Endoscopic screening can identify precancerous lesions and early-stage GC in *H. pylori*-positive patients. Our pathological results revealed higher rates of atrophy, intestinal metaplasia, active inflammation, and *H. pylori* infection in urban patients, likely because urban populations are more proactive in addressing CAG and *H. pylori* infection. Fecal occult blood is more relevant for screening for colon cancer (31) but could lead patients to upper gastrointestinal endoscopic opportunistic screening, including anemic. Urban patients are more responsive to elevated tumor markers, *H. pylori* infection, positive fecal occult blood, and anemia, which may facilitate earlier GC diagnoses.

This study has several limitations. First, it was conducted in Hangzhou, China, which limits generalizability. However, by focusing on a single region, we minimized potential confounding factors related to ethnicity, diet, culture, and GC prevalence, enhancing the reliability of our findings. Second, as a retrospective study, it was difficult to obtain comprehensive records of ESD procedures, surgeries, and prognoses for some patients with GC. Nonetheless, the available data revealed significant disparities in endoscopic examination chief complaints and outcomes between urban and rural patients, particularly in EGC diagnoses. These findings highlight the need to improve EGC screening, endoscopic surveillance, and health education in rural areas. Although the low GC diagnostic rate in rural areas is a global issue, it has received limited research attention. We hope that future studies will further explore the challenges faced by rural patients with GC.

The early diagnosis rate of GC in rural areas of Hangzhou, China, is lower than that in urban areas. Rural patients are more likely to undergo endoscopy because of symptomatic complaints such as abdominal pain, reflux, abdominal fullness, and melena, with less focus on routine health checks, endoscopic surveillance of CAG, elevated tumor marker levels, and *H. pylori* infection. Health education should be enhanced in rural areas to actively guide people at high risk of GC to undergo endoscopic screening. Regular endoscopic surveillance is recommended for patients with CAG.

## CONFLICTS OF INTEREST

**Guarantor of the article:** Liang Han, MD.

**Specific author contributions:** L.S.: performed most of the investigation, including data collection and analysis, and wrote the manuscript; Q.Y. and B.L.: designed and supervised the study including all data collection and analysis; Y.S.: assisted with the data collection and analysis; Y.H. and Y.Z.: assisted with the data collection. L.H.: designed and supervised the study including all data collection and analysis, and wrote the manuscript; All authors have read and approved the manuscript.

**Financial support:** None to report.

**Potential competing interests:** None to report.

**Study Highlights**

**WHAT IS KNOWN**

✓ The early diagnosis rate of gastric cancer in rural is lower than in urban areas.
✓ Regular endoscopic surveillance is recommended for patients with chronic atrophic gastritis.

**WHAT IS NEW HERE**

✓ Rural patients are more likely to undergo endoscopy because of symptomatic complaints.
✓ Rural patients focus less on health checks and endoscopic surveillance.
✓ Health education should be enhanced in rural areas.
